# Biochemical and molecular characteristics of leaf photosynthesis and relative seed yield of two contrasting rice cultivars in response to elevated [CO_2_]

**DOI:** 10.1093/jxb/eru344

**Published:** 2014-09-01

**Authors:** Chunwu Zhu, Jianguo Zhu, Jing Cao, Qian Jiang, Gang Liu, Lewis H. Ziska

**Affiliations:** ^1^State Key Laboratory of Soil and Sustainable Agriculture, Institute of Soil Science, Chinese Academy of Sciences, Nanjing, Jiangsu 210008, PR China; ^2^Institute of Agricultural Economics and Information, Jiangsu Academy of Agricultural Sciences, Nanjing 210014, PR China; ^3^Crop Systems and Global Change Laboratory, USDA-ARS, 10300 Baltimore Avenue, Beltsville, MD 20705, USA

**Keywords:** Elevated CO_2_, panicle, photosynthetic capacity, rice, sink, source.

## Abstract

Understanding the basis for intraspecific yield variability may be important in elucidating biological mechanisms that are associated with superior yield performance in response to projected increases in carbon dioxide concentration, [CO_2_]. Using a free-air CO_2_ enrichment (FACE) facility, two rice lines, S63 and W14, which differed consistently in their enhancement of seed yield when grown at elevated [CO_2_] in multiple field trials, were examined. To determine if the different cultivar responses were linked to changes in photosynthetic characteristics at elevated [CO_2_], spatial and temporal changes in photosynthetic stimulation and the occurrence of down-regulation, or acclimation, in relation to panicle sink development were quantified for the uppermost canopy leaves. Changes in photosynthetic capacity were determined by quantifying changes in the sink:source ratio, leaf nitrogen (N) content, the concentration and mRNA expression of the large Rubisco subunit, and changes in *V*_c,max_, the maximum ribulose bisphosphate (RuBP)-saturated rate of carboxylation. For the W14 cultivar, significant reductions in photosynthesis at the elevated, relative to ambient [CO_2_], signalling photosynthetic acclimation, were observed following panicle initiation. The observance of photosynthetic acclimation was consistent with significant reductions in N, Rubisco content and expression, and *V*_c,max_. In contrast, for the cultivar S63, elevated [CO_2_] resulted in increased spikelet number and grain weight, increased sink:source ratios, and continued stimulation of photosynthesis up to grain maturity. Overall, these data suggest that the greater response of the S63 line to elevated [CO_2_] may be associated with enhanced carbon sinks relative to sources, and the ability to maintain photosynthetic capacity during grain development.

## Summary

The basis for differential responses of rice yield to rising CO_2_ levels may be associated with temporal changes in photosynthetic capacity that sustain carbon assimilation rates during grain filling.

## Introduction

Since 1959, concentrations of atmospheric carbon dioxide, [CO_2_], have increased from 318 to ~400 ppm, and, depending on anthropogenic emission rates, may reach 1000 μmol mol^–1^ by the end of the century (Meinshausen *et al.*, 2011). Although widely recognized as a global warming gas, [CO_2_] is also the sole source of carbon for photosynthesis, and its increased availability can also directly enhance plant growth. The degree of enhancement, however, has been found to vary not only between plant species but also within a given species. Such intraspecific enhancement is of obvious interest in agricultural research because it represents a potential means to begin selection of crop cultivars that could show superior yield performance in response to rising atmospheric [CO_2_] (Tausz *et al.*, 2013).

To date, significant yield variability has, in fact, been observed for a number of crop species (Mandersheid and Weigel, 1997; Ainsworth *et al.*, 2004; Sicher *et al.*, 2010). Such variation in response to elevated [CO_2_] has also been observed for rice, with studies showing that [CO_2_] enrichment can result in dissimilarity in tiller number, leaf area, and net photosynthetic rate, as well as yield enhancement (Yang *et al*., 2006*a*, *b*; Liu *et al.*, 2008; Shimono *et al.*, 2009; Hasegawa *et al.*, 2013). However, the biological underpinnings for superior yield response to elevated [CO_2_] between cultivars is not clear. Obviously, understanding the basis for this variation would represent an important aspect for further selection and development of elevated [CO_2_]-responsive crop lines.

One potential explanation for the variable effects of increased [CO_2_] on growth and yield may be related to temporal changes in photosynthetic capacity. That is, while photosynthesis is stimulated initially by enhanced levels of [CO_2_], photosynthetic capacity may decline temporally, or acclimate, with prolonged exposure to enhanced [CO_2_] levels (Arp, 1991; Rogers and Humphries, 2000). However, whether temporal shifts in photosynthetic capacity are a factor associated with greater growth and yield enhancement among rice cultivars to elevated [CO_2_] is an open question.

In the current study, two rice lines, Shanyou 63 (S63) and Wuxiangjng 14 (W14), were examined. Previous field trials using free-air CO_2_ enrichment (FACE) have shown that these lines have a strong and weak response of seed yield at elevated, relative to ambient levels of [CO_2_], respectively (Yang *et al.*, 2006*b*; Liu *et al.*, 2008). The goal of the study was to quantify biochemical and molecular changes in photosynthetic capacity for the uppermost canopy leaves during reproductive development as a means to assess the relative contribution of leaf assimilate to panicle growth. This information, in turn, could then be used to determine which specific changes in photosynthetic capacity, if any, were associated with greater yield stimulation in response to elevated [CO_2_].

## Materials and methods

### Free-air carbon dioxide enrichment site

The study was conducted at the FACE facility located at Zhongcun village (119°42′0′′E, 32°35′5′′N), Yangzhou city, Jiangsu province, a typical Chinese rice-growing region (Zhu *et al.*, 2012). The operation and control systems for the FACE facilities were the same as those used at the Japan FACE site (Zhu *et al.*, 2009). The target [CO_2_] at the centre of the elevated FACE rings was 200 μmol mol^–1^ above ambient levels. During the 2010 season, average daytime [CO_2_] at canopy height during the experiment was 384.7 μmol mol^–1^ and 574.0 μmol mol^–1^ for the ambient and elevated FACE rings, respectively. The average temperature during the growth stage was 26.1 °C and the average relative humidity was ~93%; values typical of the growing season at this location.

### Crop cultivation

Two morphologically distinct rice (*Oryza sativa*) lines, a japonica (Wuxiangjing 14, W14) and a hybrid indica (Shanyou 63, S63) rice, were utilized in this study. Both lines had been field-tested over a 3 year period for yield enhancement to elevated [CO_2_] at the China FACE facility, with S63 consistently showing a greater (~2-fold) increase in seed yield relative to W14 (Yang *et al.*, 2006*b*; Liu *et al.*, 2008).

For the current study, seeds of each cultivar were sown at ambient [CO_2_] on 21 May 2010. The seedlings were then manually transplanted to ambient and elevated FACE rings at a density of one seedling per hill on 21 June. The spacing of the hills was 16.7 cm×25cm (equivalent to 24 hills m^–2^). Nitrogen was supplied as urea (N, 46%) and as a compound chemical fertilizer (N:P_2_O_5_:K_2_O=15:15:15%) at 25g m^–2^. N was applied as a basal dressing (30% of the total) 1 d prior to transplanting and as a top dressing at early tillering (30% of the total) and at panicle initiation (40% of the total). Phosphorus (P) and potassium (K) were applied as a compound fertilizer at 7g P_2_O_5_ m^–2^ and 7g K_2_O m^–2^; both P and K were applied as a basal dressing 1 d before transplanting.

**Fig. 1.**
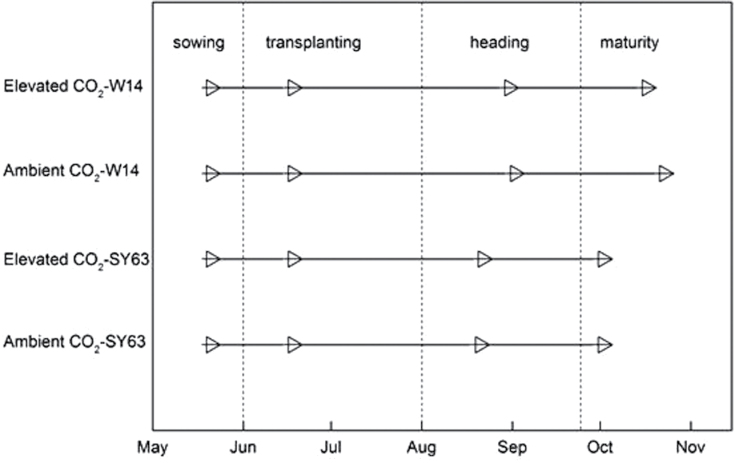
Time course and development of two rice cultivars (S63 and W14) from sowing through maturity as a function of elevated [CO_2_] for the 2010 season. Specific dates are given in the Materials and methods.

All experimental ﬁeld plots were submerged with water from 13 June to 10 July; all plots were drained between 11 July and 4 August and then ﬂooded with intermittent irrigation from 5 August to 10 d before harvest. Additional details regarding cultivation can be found in Yang *et al*. (2006*a*, *b*) and Zhu *et al.* (2009). Differences in development as a function of both cultivar and [CO_2_] concentration are shown in Fig. 1.

### Photosynthetic gas exchange and leaf area

Measurements of leaf net photosynthesis (*P*_N_, determined as CO_2_ uptake per cm^2^ leaf area) were conducted using a portable photosynthesis system equipped with an infrared gas analyser (LI-6400, LI-COR, Lincoln, NE, USA) during August 22–23, September 7–8, and September 22–23 for the S63 cultivar; and September 2–3, September 17–18, and October 3–4 for the W14 cultivar. These time periods correspond approximately to panicle initiation, mid grain fill, and end grain fill; or, using the development system of Counce *et al.* (2000), R4 (anthesis), R6 (grain depth expansion), and R8 (single grain maturity).

To produce the *P*_N_–*C*_i_ curve, the [CO_2_] was set at 580 μmol mol^–1^ (elevated leaves) or 380 μmol mol^–1^ (for ambient leaves) and then photosynthesis was determined at ambient CO_2_ concentrations of 250, 200, 150, 100, 50, 400, 600, 800, 1000, and 1200 μmol mol^–1^. Measurements were made at a saturating photosynthetic photon flux density (PPFD) of 1800 μmol m^–2^ s^–1^. Leaf temperature was set at 30 °C when CO_2_ assimilation was being measured. The *P*_N_–*C*_i_ curve was then analysed using the model of Farquhar *et al.* (1980) to predict *V*_c,max_, the maximum ribulose bisphosphate (RuBP)-saturated rate of carboxylation. Following the photosynthetic measurements, leaf area (green leaves only) for 10 of the flag leaves, and the corresponding second and third leaves below the flag leaf were determined using a leaf area detector (YMJ-A, Tuopu, China).

### Sampling and biochemical analyses

On the same day as the *P*_N_–*C*_i_ curve measurement, leaves were sampled at around midday, 11:30h to 12:30h (China standard time), when exposed to full sunlight. Leaves were then stored in liquid nitrogen until biochemical/molecular analysis. Leaf tissue nitrogen concentration was measured using an elemental analyser (PE2400 series II CHNS/O, USA).

Half of each flag leaf was used to determine the Rubisco protein content. The crude Rubisco protein extraction was based on the method of Zhu *et al.* (2012), with some modifications. The homogenates were centrifuged at 4 °C for 10min at 12 000 *g* and then the supernatant was mixed with cold acetone containing 0.07% β-mercaptoethanol. The dried protein pellets were dissolved in buffer containing bromophenol blue (62.5 mmol l^–1^ TRIS-HCl, pH 6.8, 2% SDS, 10% glycerol, 5% β-mercaptoethanol, and 0.005% bromophenol blue). The Rubisco content was determined by formamide extraction of Coomassie Brilliant Blue R-250-stained bands corresponding to the large and small subunits of Rubisco that had been separated by sodium dodecyl sulphate–polyacrylamide gel electrophoresis (SDS–PAGE) using calibration curves made from puriﬁed rice Rubisco (Nie *et al.*, 1995).

The other half of the flag leaf sample was used to quantify mRNA. Reverse transcription–PCR (RT–PCR) was carried out using 2 μg of total RNA treated with DNase I (Invitrogen). Reverse transcription was performed using SuperScript II RNase H- (Invitrogen) and oligo(dT)_23_ primers (Sigma-Aldrich). PCR was performed using cycles of 94 °C for 30 s, 55 °C for 30 s, and 72 °C for 60 s, followed by a ﬁnal extension at 72 °C for 7min. Quantitative RT–PCR was carried out on a Thermal Cycler Dice TP800 system (Takara Bio) using SYBR premix Ex Taqmixture (Takara) with cycles of 95 °C for 5 s and 60 °C for 30 s. *Rice ubiquitin 1* (*Rubq1*; AK121590) was used as an internal standard. The primers *rbcL*: F-5′ CTTGGCAGCATTCCGAGTAA 3′ and R-5′ CAACGGGCCGATGTGATA 3′, were used in the PCR study. The primer sets were tested by dissociation curve analysis and veriﬁed for the absence of non-speciﬁc ampliﬁcation.

### Determination of the sink:source ratio

Changes in sink:source ratios were determined using a source manipulation experiment whereby source leaves were shaded. Eight culms (tillers) from each [CO_2_] treatment and cultivar which had recently (<24h) headed (i.e. initiated a panicle) were chosen. Four shading treatments (eight tillers per treatment, [CO_2_], and cultivar) were used that continued until physiological maturity: (i) flag leaves only; (ii) second leaves only; (iii) third leaves only; or (iv) all leaves (flag, second, and third). The shading material was aluminium foil perforated with 1mm diameter holes at least 15mm apart. This was done to prevent the accumulation of ethylene and water vapour. Panicle weight was determined at the end of each shading treatment. Sink:source ratios were determined as the ratio of the panicle biomass to that of unshaded leaf area (expressed as mg of panicle yield per cm^2^ of leaf area).

### Yield components

The grain yield and yield components were measured according to Liu *et al.* (2008). At maturity, all plants from a 1 m^2^ area (excluding plants on the borders) were cut at ground level and separated into vegetative and reproductive components. Collected grains (seed) were soaked in 1.00 speciﬁc gravity tap water and the numbers of sunken and ﬂoated grains were counted to determine the ﬁlled spikelet percentage. The dry weight of ripened (sunken) grains was determined after oven-drying at 80 °C for 72h. Grain yield and single-grain mass were expressed on a 14% moisture content basis.

### Statistical analysis

The field experiment was a completely randomized design with three replicates. To test for significant differences, analysis of variance (ANOVA) with [CO_2_] as the main plot, cultivars as subplots, and stage of growth as a split plot was used. Statistical calculations were carried out using SPSS statistical software (SPSS 19.0, SPSS Inc., Chicago, IL, USA).

## Results

### Total yield and yield components

Previous multiyear field trials have shown a consistently greater yield response of S63 relative to the W14 cultivar, including the 2010 trial (Table 1A). Consequently, there is a significant interaction between [CO_2_] and cultivar. Overall, in regard to yield variables, S63 had a lower panicle number than W14, but a higher spikelet number per panicle, a higher weight per grain, and a higher filled spikelet ratio (Table 1).

**Table 1:** Averages for grain yield and yield components for two rice cultivars, S63 and W14, grown *in situ* at ambient and elevated CO_2_ concentrations using FACE methodology

**(A) Long-term (3 year) data combined from Liu *et al.* (2008) and Yang *et al*. (2006*a*, *b* ).**						
**Variety**	**Variety[CO_2_]**	**VarietyPanicle number (m^–2^)**	**VarietySpikelets per panicle**	**VarietyFilled spikelet ratio (%)**	**VarietyWeight per grain (mg)**	**VarietyYield (g m^–2^)**
S63	Ambient	237.6	162.1	77.8	30.0	986.7
	Elevated	259.6	181.3	81.1	31.4	1281.0
	% Change	9.2	11.8	4.2	4.6	29.8
W14	Ambient	292.1	154.5	66.0	28.1	996.7
	Elevated	343.0	141.7	67.8	28.4	1120.0
	% Change	17.4	–8.3	2.7	1.2	12.4
**Variety(B) Data from 2010 for the same cultivars and location**						
**VarietyVariety**	**Variety[CO_2_]**	**VarietyPanicle number (m^–2^)**	**VarietySpikelets per panicle**	**VarietyFilled spikelet ratio (%)**	**VarietyWeight per grain (mg)**	**VarietyYield (g m^–2^)**
S63	Ambient	258.3	162.9	77.9	30.5	999.7
	Elevated	280.9	176.9	83.3	31.6	1308.9
	% Change	9.4	8.5	6.9	3.6	30.9
W14	Ambient	313.2	147.5	70.3	28.0	927.1
	Elevated	356.7	138.5	73.1	28.6	1065.3
	% Change	13.9	–6.2	4.0	2.1	15.0
Variable		Panicle number (m^–2^)	Spikelets per panicle	Filled spikelet ratio (%)	Weight per grain (mg)	Yield (g m^–2^)
[CO_2_]		*	NS	NS	NS	**
Cultivar		*	*	*	*	*
[CO_2_]×cultivar		NS	*	NS	NS	*

% Change was determined as (elevated–ambient)/ambient×100%.

ANOVA was determined with [CO_2_] as the main plot, and rice cultivars as subplots. **P≤0.01; *P≤0.05; NS, not significant, *P*>0.05

### Photosynthetic acclimation

**Fig. 2.**
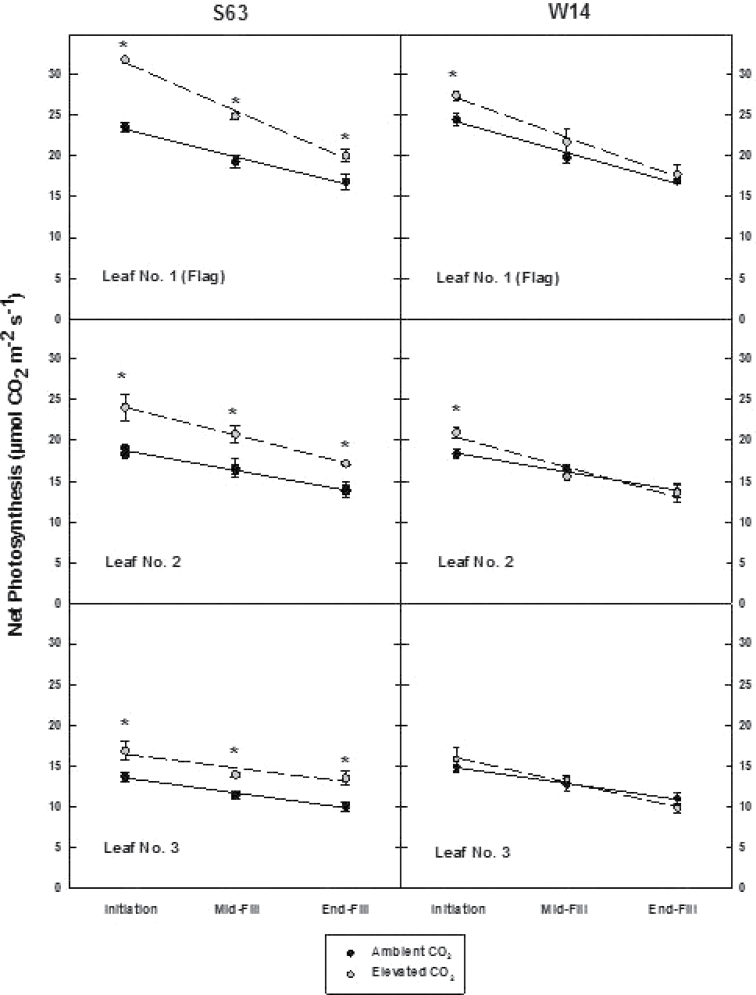
Changes in net photosynthesis (μmol m^–2^ s^–1^) of the three uppermost canopy leaves for two rice cultivars (S63 and W14) grown at ambient and elevated [CO_2_] *in situ* using a FACE facility. Measurements were taken at three stages of reproductive development. These correspond to panicle initiation, mid grain fill, and end grain fill; or R4 (anthesis), R6 (grain depth expansion), and R8 (single grain maturity) using the system of Counce *et al.* (2000). Asterisks indicate a significant difference at a given stage for either cultivar as a function of [CO_2_] treatment. **P<0.01; *P<0.05; bars are ±SD.

There was a temporally consistent increase in photosynthetic rate with elevated (relative to ambient) [CO_2_] from heading (floral initiation) through grain fill for the uppermost three leaves for the S63 cultivar (left column, Fig. 2). In contrast, while elevated [CO_2_] did stimulate the leaf photosynthetic rate for the flag leaf and the second leaf at heading, no further effect of [CO_2_] on leaf photosynthesis through grain fill was observed (right column, Fig. 2) for the W14 cultivar.

### Sink:source ratio

**Fig. 3.**
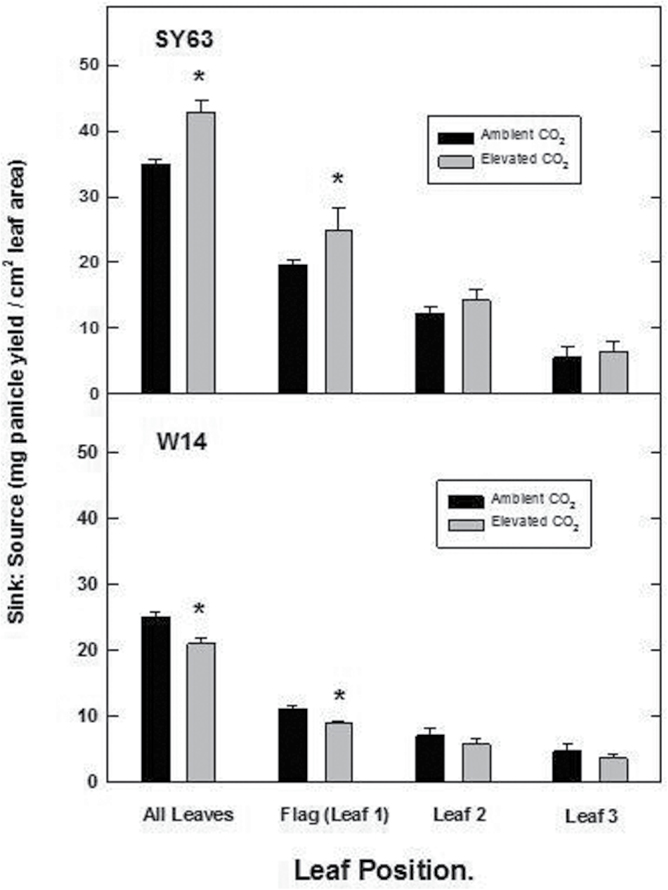
The carbon source:sink ratio (mg of panicle to cm^2^ of leaf area) for the uppermost three leaves (averaged and individual) for the rice cultivars SY63 and W14 grown at ambient and elevated [CO_2_]. The ratio was determined from shading of individual leaves (source) and measurement of panicle biomass (sink) from post-anthesis through individual grain maturity (R4–R8 using the system of Counce *et al.*, 2000). Asterisk(s) indicate a significant differences for a given cultivar as a function of [CO_2_]. See the Materials and methods for additional details. **P<0.01; *P<0.05; bars are ±SD.

The ratio of carbon sinks (panicle biomass) to sources (green leaf area) was significantly different as a function of cultivar (Fig. 3). When averaged from initiation through grain filling, elevated [CO_2_] significantly enhanced this ratio for the flag leaf and the uppermost three leaves of the S63 cultivar (all leaves). In contrast, a significant decrease in the sink:source ratio was observed for the flag leaf and uppermost three leaves for the W14 line. Overall, this suggests a greater efficiency of photosynthetic response, (i.e. more panicle biomass is supported by the same amount of leaf area) at elevated [CO_2_] for the S63 relative to the W14 cultivar. As a consequence, there was a significant cultivar×CO_2_ interaction for sink:source ratio (Fig. 3).

### Leaf photochemistry

**Fig. 4.**
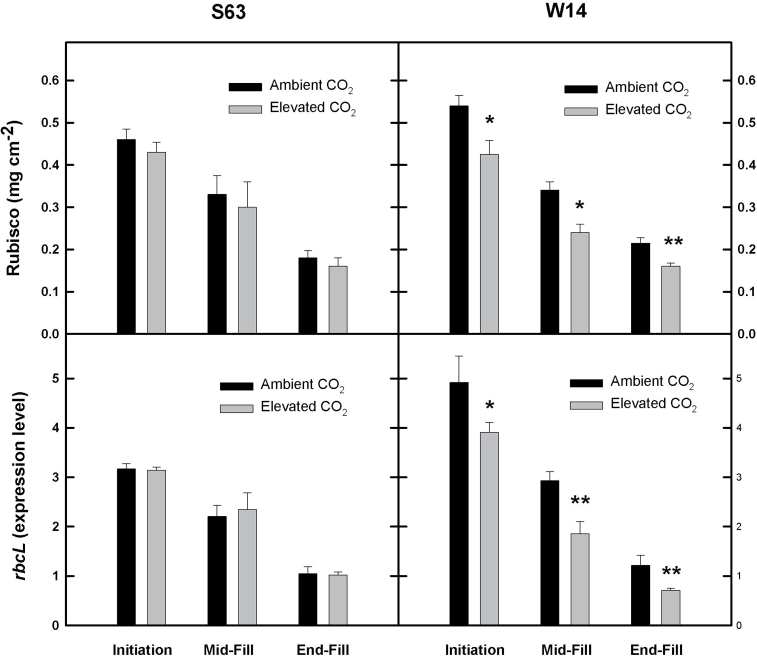
Average (±SD) Rubsico content (mg cm^–2^) and mRNA expression of the large Rubisco subunit (*rbcL*) of the flag leaf for two rice cultivars, S63 and W14, grown at ambient and elevated [CO_2_] *in situ* using a FACE facility. Measurements were taken as described in Fig. 2. Asterisks indicate a significant difference at a given stage for either cultivar as a function of [CO_2_] treatment. **P<0.01; *P<0.05; bars are ±SD.

As the primary enzyme for CO_2_ fixation, changes in photosynthetic capacity should reflect both Rubisco content and its gene expression. For both cultivars, Rubisco content and the extent of gene expression for the large subunit declined during grain filling for the flag leaf, consistent with the temporal decline in photosynthesis observed in Fig. 2 (Fig. 4). However, for S63, [CO_2_] did not alter the rate of decline, or content/gene expression. In contrast, significant declines in both Rubisco content and gene expression were noted for the W14 cultivar from initiation until the end of grain fill (Fig. 4).

Changes in Rubsico concentration should also be indicative of overall changes in nitrogen (i.e. protein) content. For the current experiment, in response to elevated [CO_2_] small but significant reductions in leaf nitrogen were observed for the flag leaf of the S63 cultivar at heading and the end of grain fill; for the second leaf throughout grain filling, and for the third leaf at the end of grain fill (Table 2). For the W14 cultivar, reductions in N were observed at panicle initiation for all of the uppermost leaves at elevated [CO_2_], and for the flag and third leaf at the end of grain fill. Overall, when averaged for all leaves and measurement times for a given cultivar, elevated [CO_2_] resulted in a 7.6% and 27% decline in leaf N for the S63 and W14 cultivar, respectively; consistent with the overall decline observed for Rubisco content (Fig. 4).

**Table 2.** *V*_c,max_ and N content for the flag, second, and third leaves for the rice cultivars, S63 and W14, grown under two different [CO_2_] (ambient and elevated) Means were determined for three replicate elevated and ambient [CO_2_] plots during three stages of reproductive development; these correspond to panicle initiation, mid grain fill, and end grain fill; or R4 (anthesis), R6 (grain depth expansion), and R8 (single grain maturity) using the system of Counce *et al.* (2000).

**Cultivar**	**[CO_2_]**	**Stage**	***V*_c,max_ (μmol m^–2^ s^–1^)**			**N (μg cm^–2^)**		
			**Flag**	**Leaf 2**	**Leaf 3**	**Flag**	**Leaf 2**	**Leaf 3**
S63	Ambient	Initiation	123.7	105.1	70.6	163.7	144.3	117.3
	Elevated	Initiation	124.2	102.9	67.6	152.1*	132.2*	113.0
	Ambient	Mid-Fill	100.3	79.9	49.3	131.0	115.3	93.7
	Elevated	Mid-Fill	100.6	77.2	50.7	121.7	105.7*	90.3
	Ambient	End-Fill	81.0	71.1	55.2	112.3	95.3	74.1
	Elevated	End-Fill	81.0	70.1	53.1	100.1*	85.3*	67.3*
W14	Ambient	Initiation	129.2	105.0	78.3	175.3	153.3	124.3
	Elevated	Initiation	111.7*	92.4*	65.3*	148.2*	127.3*	104.3*
	Ambient	Mid-Fill	103.6	82.9	52.3	140.7	120.7	110.0
	Elevated	Mid-Fill	87.7*	65.4*	40.2*	119.7	102.6	90.0
	Ambient	End-Fill	80.5	57.3	47.3	112.3	86.2	75.7
	Elevated	End-Fill	64.5*	45.1*	32.3*	84.6*	66.7*	51.7*
**ANOVA**								
			***V*_c,max_ (μmol m^–2^ s^–1^)**			**N (μg cm^–2^)**		
			**Flag**	**Leaf 2**	**Leaf 3**	**Flag**	**Leaf 2**	**Leaf 3**
Cultivar			**	**	**	NS	*	**
[CO_2_]			**	**	**	**	**	**
Stage			**	**	**	**	**	**
Cultivar×[CO_2_]			**	**	**	**	**	**
Cultivar×stage			NS	NS	**	**	**	**
[CO_2_]×stage			NS	**	NS	NS	NS	NS

In the first part of the table, an asterisk indicates a significant difference as a function of [CO_2_] for a given cultivar and growth stage.

ANOVA was determined with [CO_2_] as the main plot, and rice cultivars as subplots. **P ≤0.01; *P ≤0.05; NS, not significant, *P*>0.05.

Consistent with the change in Rubisco content and gene expression, *V*_c,max_, the maximum RuBP-saturated rate of carboxylation, declined from panicle initiation through grain fill for both rice lines (Table 2). No change in *V*_c,max_ was observed as a function of [CO_2_] for any of the growth stages for the S63 cultivar (Table 2). However, for W14, *V*_c,max_ significantly decreased at elevated, relative to ambient [CO_2_] for all leaf positions, and for all times during grain fill. In addition, for both N and *V*_c,max_, the third leaf position for W14 showed a significant trend for a greater decline as a function of time and canopy depth. Not surprisingly there was a significant relationship (*P*<0.0001) between *V*_c,max_ and N when leaf position and crop developmental stage were pooled within CO_2_ treatments for both cultivars. Elevated [CO_2_] increased the photosynthetic rate, and decreased the N concentration, resulting in an increase in N use efficiency for both cultivars (data not shown). Significant interactions were observed for [CO_2_] and cultivar for all leaf positions in regard to N content and *V*_c,max_ (Table 2).

## Discussion

It has been suggested that assimilate distribution between sources (e.g. leaves) and sinks (e.g. seed) of carbon in response to enhanced [CO_2_] may be a crucial factor in the ability of a cultivar to respond reproductively (Arp, 1991). For example, if high temperatures or low N limited sink development (e.g. floral sterility, reduced tiller production), then any stimulatory response to elevated [CO_2_] may be negated as lack of sinks could result in a reduction in photosynthetic capacity (e.g. Lin *et al.*, 1997; Kim *et al.*, 2003). Conversely, at optimal N and temperature, [CO_2_]-induced increases in sink development (e.g. early tiller development, increased panicle size) may significantly enhance seed yield of rice by stimulation of photosynthetic capacity over longer time periods (Shimono and Okada, 2013; Ziska *et al.*, 2014).

Some experiments suggest that allocation of additional carbon for increased sink development, through either panicle size or increased tiller production, may be associated with yield stimulation at elevated [CO_2_] (e.g. Shimono *et al.*, 2009; Shimono, 2011; Hasegawa *et al.*, 2013; Shimono and Okada, 2013). While allocation between sources and sinks in response to elevated [CO_2_] has been characterized at the whole-plant level as a function of cultivar, temporal quantification of leaf photosynthesis (e.g. acclimation or down-regulation) associated with such morphological patterns (i.e. changes in source:sink ratios) between cultivars is lacking.

For the present study, a ‘strong’ and ‘weak’ seed yield response to elevated [CO_2_] had been observed between S63 and W14 as determined via multiyear field trials at the FACE facility during the 2000s (Yang *et al.*, 2006*b*; Liu *et al.*, 2008), and again in 2010 (the current study). Overall, these data indicated a consistently greater (~2-fold) response of seed yield for the S63 relative to the W14 rice cultivar in response to elevated [CO_2_]. This variation, in turn, provided an opportunity to quantify temporal changes to the biochemical and molecular characteristics of photosynthesis for the flag and uppermost canopy leaves, and to determine if these changes in photosynthetic capacity were coherent with the observed stimulation of seed yield between cultivars.

The current study demonstrates that differences in [CO_2_]-induced seed yield stimulation between S63 and W14 are, in fact, consistent with significant temporal and spatial differences in photosynthetic capacity. For the S63 cultivar, the greater yield response was linked with continued photosynthetic stimulation at the elevated relative to the ambient [CO_2_] treatment throughout panicle and grain development for all upper canopy leaves. Continued stimulation may, in turn, be associated with increases in the carbon sink:source ratio and subsequent maintenance of Rubisco content and gene expression, as well as *V*_c,max_ at elevated, relative to ambient [CO_2_] for this cultivar. Conversely, for the W14 cultivar, a weak yield response to elevated [CO_2_] was associated with photosynthetic acclimation following panicle initiation for the uppermost leaves; a decline in the sink:source ratio and significant reductions in Rubisco and *V*_c,max_ during grain development. In addition, for this cultivar, the extent of acclimation may be a function of leaf position, with leaves lower in the canopy (i.e. the third leaf) showing a greater degree of down-regulation. Greater down-regulation of photosynthesis has been reported previously for lower canopy leaves in both wheat (Osborne *et al.*, 1998) and rice (Seneweera *et al.*, 2011) in response to elevated [CO_2_].

While there are clear differences in the degree of photosynthetic stimulation and the occurrence of acclimation between the two cultivars, the molecular/genetic basis underlying these cultivar differences is not entirely clear. Such genetic differences reflect cultivar ‘choices’ in regard to specific organ development (e.g. spikelets per panicle), N remobilization, Rubsico production, onset of senescence, etc, when grown at elevated [CO_2_]. Differential cultivar utilization of [CO_2_] for source and/or sink development is likely to be genetically based, and deserves additional study in the context of maintenance of photosynthetic capacity and subsequent yield stimulation between cultivars.

Overall, the occurrence of photosynthetic acclimation in rice and other cereals in response to elevated [CO_2_] is known and characterized (Lin *et al.* 1997; Zhu *et al.* 2012); however, to the authors’ knowledge, quantification of such changes has not been related to intraspecific yield variations among rice lines to elevated [CO_2_]. Previous work with soybean (Ziska *et al.*, 1998) indicated that the relative enhancement of either vegetative or reproductive growth at elevated CO_2_ was not, in fact, correlated with changes in the absolute or relative increase in single leaf photosynthetic rate among cultivars at elevated [CO_2_]. However, for rice, in contrast to soybean, reproductive development may be more closely aligned to assimilate produced and transported from the flag leaf. Hence [CO_2_]-induced changes in photosynthetic capacity, particularly for the flag leaf and upper canopy during grain filling, may be more relevant to stimulation of seed yield.

Overall, while the current study demonstrates a coherent link between elevated [CO_2_]-induced increases in seed yield, and biochemical and molecular changes that improve photosynthetic capacity through grain filling, it is only reflective of two cultivars. Nevertheless, the current data do illustrate that for rice, photosynthetic capacity in response to source:sink development may be a crucial mechanism underlying intraspecific yield sensitivity to elevated [CO_2_]. However, additional rice lines with known differential seed yield sensitivity to projected increases in atmospheric [CO_2_] need to be examined in the context of differential source:sink development to confirm and refine these observations.
